# Datasets for corporate governance index of Jordanian non-financial sector firms

**DOI:** 10.1016/j.dib.2020.105603

**Published:** 2020-04-22

**Authors:** Marwan Mansour, Hafiza Aishah Hashim, Zalailah Salleh

**Affiliations:** Faculty of Business Economics and Social Development, Universiti Malaysia Terengganu, 21030, Kuala Terengganu, Terengganu, Malaysia

**Keywords:** Corporate governance, Index, Non-financial firms, Jordan

## Abstract

This article covers comprehensive data on firm-level corporate governance practices as imposed by the Jordan Securities Commission (JSC). The study includes panel data for 95 non-financial Jordanian listed firms (industrial and service sector) in Amman Stock Exchange (ASE). The time frame used for this study is from 2012 to 2017. Data presented were extracted from the annual reports of each firm. The annual reports had been downloaded from the official website of the ASE. The data can be used easily by the researcher to develop and calculate a corporate governance index that involves thirty-two internal governance attributes and is comprised of three equally weighted sub-indices. The first sub-index which is “Disclosure and Transparency” consists of 15 unique attributes. While the second sub-index, “Board Effectiveness and Composition” consists of 9 unique attributes. The last sub-index which is “Shareholders Rights” consists of 8 unique attributes. Thus, the un-weighted corporate governance index has an important feature that is easily replicated and modified, enabling the researcher to rate firms based on an aggregate index score or by using the sub-indices score also.

**Specifications Table**

**Table:** 

Subject	Accounting
Specific subject area	Corporate governance
Type of data	Table (Excel file)
How data were acquired	The dataset was manually collected from the annual reports of firms by using content analysis and annual reports downloaded from the official website of ASE.
Data format	Filtered
Parameters for data collection	Non-financial firms sector listed in ASE and had available information for the current study during the entire study period. The non-financial firms sharing the same requirements as imposed by the JSC, for instance, the corporate governance code. This study excluded the financial firms' sector due to variations in the regulatory framework of non-financial sector firms.
Description of data collection	The data were hand-collected from the annual reports of each firms for the pertaining years that were published at the official website of ASE. Depending on content analysis of 95 non-financial firms annual reports for six years observations (2012-2017).
Data source location	Jordan
Data with this article.	The data are attached within this article

## Value of the data

• The dataset includes unique attributes and comprehensive corporate governance practices in the Jordanian non-financial sector in the absence of a systematic technique or standardized system to measure the compliance of firms with the country's corporate governance code.
• The data could be useful for researchers, investors, and firms to assess firms’ compliance with corporate governance provisions.
• The data can be useful in constructing a corporate governance index for assessing and ranking non-financial firms' compliance with corporate governance provisions. This assessment can be used by investors to make comparisons between firms so they can make appropriate investment decisions.
• The data can be valuable for researchers to explore the relationship between corporate governance indices and financial/operating performance of firms.
• The holistic evaluation of corporate governance by using governance indices offer complementary views.

## 1 Data description

The cross-sectional data encompass of 570 year-observations of 95 non-financial firms recorded in the ASE database for six years from 2012 to 2017. We also retrieved some data from the Securities Depository Center (SDC). These datasets can be used to develop a corporate governance index based on a binary scale of attributes in Table 2. Thus, the industrial sector firms of 49 firms are distributed over 9 segments, while the services sector firms containing 46 firms are distributed over 8 segments as shown in Table 1.

**Table 1:** Descriptive statistics for the frequency of governance attributes in non-financial firms’ segments.

	Industrial sector firms	No. of firms	Freq. (Segments)	% Industrial sector
1	Pharmaceutical and Medical Industries	4	600	8.17
2	Chemical Industries	8	1177	16.02
3	Food and Beverages	10	1510	20.555
4	Tobacco and Cigarettes	2	306	4.165
5	Mining and Extraction Industries	12	1810	24.64
6	Engineering and Construction	6	871	11.856
7	Electrical Industries	3	462	6.29
8	Textiles, Leathers and Clothing's	3	433	5.894
9	Printing & Packaging	1	177	2.41
	**Total industrial firms**	**49**	**7346**	**100%**

	Services sector firms	No. of firms	Freq. (Segments)	% Services sector

1	Health Care Services	4	614	8.829
2	Educational Services	6	926	13.314
3	Hotels and Tourism	9	1350	19.41
4	Transportation	9	1314	18.892
5	Technology and Communication	2	314	4.515
6	Media	1	149	2.142
7	Utilities and Energy	5	797	11.46
8	Commercial Services	10	1491	21.438
	**Total services firms**	**46**	**6955**	**100%**
	**Total for all segments**	**95**	**14301**	

Table 1 shows the frequency of governance elements in non-financial Jordanian firms. It also demonstrates the frequency based on main sectors which had been classified to industrial sector firms that include segments (Pharmaceutical & Medical Industries, Chemical Industries, Food & Beverages, Tobacco & Cigarettes, Mining & Extraction Industries, Engineering & Construction, Electrical Industries, Textiles, Leathers & Clothing's, and Printing & Packaging) and services firms that include segments (Health Care Services, Educational Services, Hotels & Tourism, Transportation, Technology & Communication, Media, Utilities & Energy, and Commercial Services).

## 2 Experimental design, materials, and methods

We collected the 32 governance elements for each non-financial firm individually. All governance elements in Table 2 were converted to a binary variant. The elements were coded as “1” if a firm has the attribute and “0” otherwise. The dichotomous variables were used for constructing the corporate governance index for non-financial Jordanian firms. Following Al Malkawi et al. [1], each attribute in the index is based on a binary scale taking a value of (1 or 0). The value of 1 indicates the existence/ commitment of the attribute and 0 indicates the absence/ non-commitment. The final maximum value obtained is 32. This value is assigned to those firms that comply with all the attributes and later on converted (scaled) to a percentage. Finally, these data for all firms were merged to build a combined datasheet. The binary scoring is considered suitable for measuring the corporate governance index, due to the nature of each attribute that had been used to construct the index. In addition, Nerantzidis [2] confirms that binary scoring is considered excellent in research that intended to present firms’ compliance scores.

**Table 2:** Elements used to construct the corporate governance Index in Jordanian non-financial firms*.

A.	**Disclosure and Transparency (D & T)**	Freq.	Mean	Min	Max

1.	Firms have a website to disclose related information such as annual reports and financial statements	149	0.261	0	1
2.	The availability of the firm's annual reports to the public	570	1	0	1
3.	The firm reports comply with the International Financial Reporting Standards (IFRS)	570	1	0	1
4.	Firms disclose about annual reports in the English language	191	0.335	0	1
5.	The firm employs one of the well-known Big-4 auditor firms	232	.407	0	1
6.	Is the auditor's report clean	491	.861	0	1
7.	The annual reports must specify any potential conflicts of interest like issues regarding the related party transactions	564	.989	0	1
8.	The firm provides details (report) on the corporate social responsibility	360	.632	0	1
9.	The firm reveals the benefits and remunerations of the board members	570	1	0	1
10.	The firm reveals the benefits and remuneration of the Senior Executive Management	568	.996	0	1
11.	The firm reveals the qualifications of the Senior Executive Management	568	.996	0	1
12.	Information related to risk management is available in the annual report	564	.989	0	1
13.	The firm has a corporate governance report	442	.775	0	1
14.	The firm provides details about the credit rating	13	.023	0	1
15.	Availability of details relating board of directors’ meetings (Board activity) and attendance	400	.702	0	1

B.	**Board Effectiveness and Composition (BE & C)**				

16.	The CEO and board chairman are different persons	499	.875	0	1
17.	The firm has an audit committee	526	.923	0	1
18.	The firm has a nomination committee, a remuneration committee	295	.517	0	1
19.	The firm has a majority of non-executive directors	464	.814	0	1
20.	The board size is between 5 and 13	563	.988	0	1
21.	The firm has revealed the qualifications of the board members in the annual report	566	.993	0	1
22.	The firm revealed shares owned by directors’ members	564	.989	0	1
23.	The firm revealed shares owned by Senior Executive Management	562	.986	0	1
24.	The independent directors form 1/3 of the total	231	.405	0	1

C.	**Shareholders’ Rights (SR)**				

25.	Offering the detailed information about the shareholders on firm's website and/or ASE website	570	1	0	1
26.	Providing the reports of the shareholders' meetings	570	1	0	1
27.	Availability of the national and foreign shareholding percentage on the firm's website and/or financial market website	570	1	0	1
28.	Availability of the authorized percentage of shareholdings by foreign shareholding	570	1	0	1
29.	The firm permits cumulative voting for the election of directors	240	.421	0	1
30.	The firm has complaint options	121	.212	0	1
31.	The dividend declarations are available to the shareholders	568	.996	0	1
32.	The market price of share is available to the shareholders	570	1	0	1

⁎ These elements used to construct the Corporate Governance Index for 95 Jordanian non-financial firms during the period (2012-2017).

## Appendix Supplementary materials

Image, application 1

Image, application 2
